# Biophysical Reviews’ “Meet the Councilor Series”—a profile of Peter Pohl

**DOI:** 10.1007/s12551-021-00897-4

**Published:** 2021-11-23

**Authors:** Peter Pohl

**Affiliations:** Institute of Biophysics, Johannes Kepler University, Linz, Austria

## Abstract

It is my pleasure to write a few words to introduce myself to the readers of *Biophysical Reviews* as part of the “Meet the Councilor Series.” Currently, I am serving the second period as IUPAB councilor after having been elected first in 2017. Initially, I studied Biophysics in Moscow (Russia) and later Medicine in Halle (Germany). My scientific carrier took me from the Medical School of the Martin Luther University of Halle-Wittenberg, via the Leibniz Institute for Molecular Pharmacology (Berlin) and the Institute for Biology at the Humboldt University (Berlin) to the Physics Department of the Johannes Kepler University in Linz (Austria). My key research interests lie in the molecular mechanisms of transport phenomena occurring at the lipid membrane, including (i) spontaneous and facilitated transport of water and other small molecules across membranes in reconstituted systems, (ii) proton migration along the membrane surface, (iii) protein translocation, and (iv) bilayer mechanics. Training of undergraduate, graduate, and postdoctoral researchers from diverse academic disciplines has been—and shall remain—a consistent part of my work.


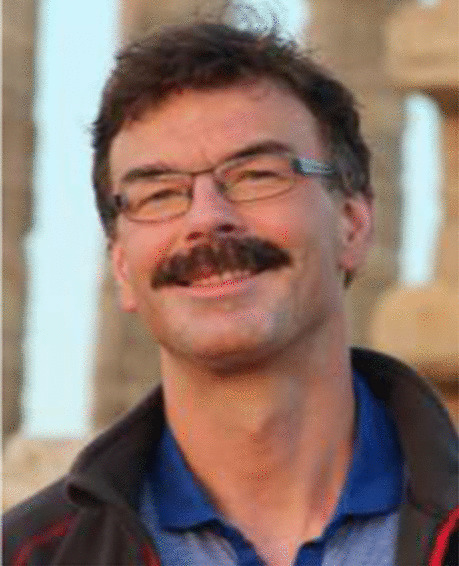


## Training

My scientific carrier started in East Germany, at the Martin Luther University of Halle-Wittenberg. I had only been working there for a month when the Berlin Wall fell. The subsequent unification of Germany had far-reaching consequences for science. The new federal state of Saxony-Anhalt took over the hundreds of years old Martin Luther University. However, its government declined to do the same with the employed professors. As a consequence, 80 out of 83 professors of the Faculty of Medicine had to step down. It took many years until their positions were filled again. I was lucky to attract extramural funding from the Deutsche Forschungsgemeinschaft (DFG). Only about a year after receiving my MD, I was awarded a grant that enabled me to pursue my research. Equally important was the continuous support from my wife, Elena Pohl, who initially worked with me in the same research department (see, e.g., Pohl et al. 2000).

With DFG’s unceasing support, I built up my research team at the Martin Luther University. However, the abrupt closure of my “scientific home,” the Institute of Medical Physics and Biophysics, posed a new challenge. Again, the DFG paved a way out of the dilemma. Endowed with DFG’s Heisenberg Fellowship, I moved to the Leibniz Institute of Pharmacology in Berlin with some of my group members. Its director Walter Rosenthal warmly welcomed me, and the scientists working there, especially Burkhard Wiesner, supported and encouraged me. Subsequently, I accepted an interim position as Professor for Biophysics at the Institute for Biology of the Humboldt University of Berlin. In 2004, I moved on to Linz (Austria), where I was appointed full professor for Biophysics in the Physics Department of the Johannes Kepler University Linz. Under my leadership, the Institute of Biophysics has grown considerably and has become one of the largest institutes at the Johannes Kepler University. It now has two departments, whose heads take turns leading the institute.

In addition to my work in the IUPAB’s council, I am serving as a member of the editorial boards of Biophysical Reviews, Biomolecules, and Scientific Reports. I was the founding chair of the youngest of the three sections of the German Biophysical Society. In this capacity, I have organized the first section meetings. Since then, I have organized and helped organizing further scientific conferences and workshops, such as the 2021 meeting of the European Biophysical Societies’ Association (EBSA) in Vienna.

## Spontaneous membrane transport of small molecules

I have long been interested in the spontaneous diffusion of small molecules across membranes (for a review, see Hannesschlaeger et al. (2019)). Despite its apparent simplicity, the spontaneous transport of small molecules is still a matter of debate (Saparov et al. 2006; Missner et al. 2008a; Boron et al. 2011). With Yuri N. Antonenko from the Moscow Lomonosov University, I have introduced scanning electrochemical microscopy to quantify small molecule transport through planar lipid bilayers (Antonenko et al. 1993; Pohl et al. 1993). This technique exploits the fact that the anionic form of weak acids gets preferably protonated upon membrane partitioning. As a consequence, pH increases in the aqueous layers adjacent to the membrane. Proton release from buffer molecules and the ensuing buffer diffusion counteract the pH change. Eventually, a steady-state pH profile develops in the immediate membrane vicinity. Recording the proton concentration as a function of the distance to the membrane by scanning pH-sensitive microelectrodes allows quantification of the transmembrane acid flux. The same approach works at the acid receiving side of the membrane or for the permeability assessment of weak bases (Antonenko et al. 1997). The determination of the intrinsic membrane permeabilities of H_2_S (Mathai et al. 2009), CO_2_ (Missner et al. 2008b), or vitamin C (Hannesschlaeger and Pohl 2018) represents valuable applications. The method can easily be adapted to permeability measurements on cell monolayers (Zocher et al. 2012) or the characterization of near-membrane unstirred layers (Pohl et al. 1998).

## Membrane transport of water

**Fig. 1:**
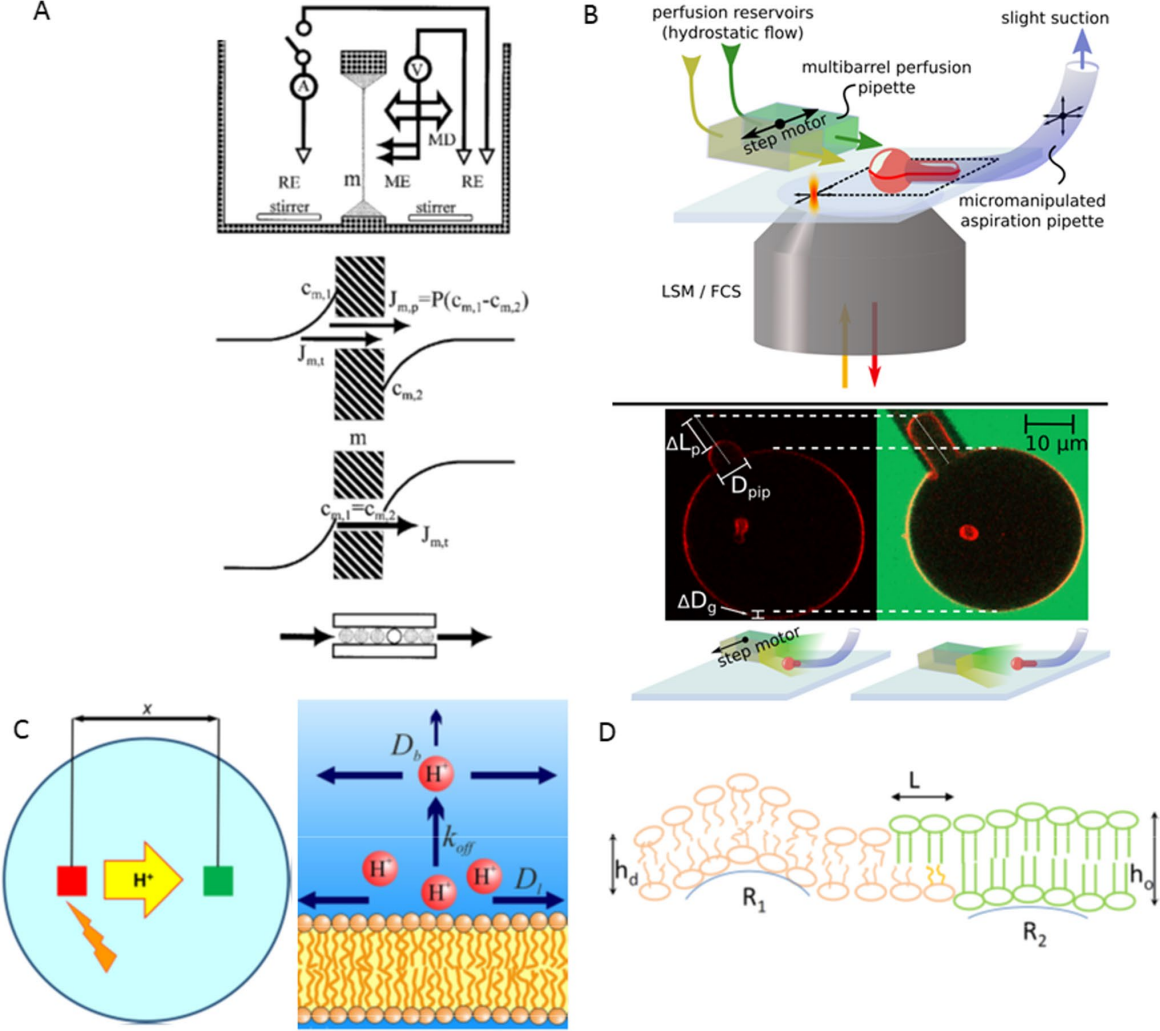
Different research areas. **A** Scanning electrochemical microscopy for water flux measurements. A picoampermeter measures the current across the cation permeable membrane using reference electrodes (RE). Osmotic water flow up-concentrates both membrane permeable and impermeable cations on one side of the membrane and depletes them on the other side (near-membrane concentrations, c_m,1_ and c_m,2_). To monitor these changes, a hydraulic microdrive (MD) moves a double-barreled microelectrode (ME) perpendicular to a planar lipid bilayer (m). A transmembrane difference in the permeable cation’s bulk concentrations served to level the concentrations at the two membrane/water interfaces, thereby rendering possible the quantitative assessment of true solvent drag. That is, water molecules push the cations (flux component J_t_) through the roughly one-water molecule wide channel, while no net flux due to an ion concentration gradient (flux component J_m,p_) occurs. The concentration distribution of the impermeable ion allows the calculation of the total water flux. Reprinted from Pohl and Saparov (2000) with permission from Elsevier. **B** Measurements of unitary water permeability by micropipette aspiration. An inverse confocal laser scanning microscope allows the observation of fluorescently labeled aspirated giant unilamellar vesicles (GUV). GUV’s protrusion length changes when the perfusion pipette switches position to expose the adjacent GUV to a hyperosmotic, carboxyfluorescein-containing buffer solution. Counting the reconstituted water channels by fluorescence correlations spectroscopy allows obtaining the unitary water permeability. Reprinted from Boytsov et al. (2020). **C** The left panel shows the experimental settings. We monitor proton surface diffusion by photo-releasing caged protons from a membrane spot (red square) and monitoring their arrival in a distant patch (green square) as a change in fluorescence intensity of a lipid-anchored pH sensor. The right panel outlines our nonequilibrium model for proton migration along the membrane surface. Proton diffusion within the confinement of the membrane hydration layers does not involve titratable residues on the surface. Proton surface-to-bulk release is irreversible. Both panels are from Weichselbaum et al. (2017). **D** Schematic of the mechanisms leading to the registration of lipid domains from the two membrane leaflets. Ordered lipids (green lipids) phase separate from disordered lipids (orange). Both (i) the line tension around ordered domains and (ii) membrane undulations induce leaflet cross-talk. The line tension occurs as a result of the hydrophobic mismatch between the lipids in the thicker (bilayer thickness *h*_o_) ordered domain and the lipids in the thinner (bilayer thickness *h*_d_) disordered domain. Deformations of border lipids minimize the access of water to the hydrophobic acyl chains. A two-step transition from *h*_o_ to *h*_d_, i.e., a small shift *L* between the edges of the ordered domains in the two leaflets saves deformational energy (Galimzyanov et al. 2015). Softer, disordered lipids and stiffer ordered domains populate areas with different monolayer curvatures (R_1_, R_2_). Reprinted from Friedman et al. (2018)

Together with my friend and colleague Sapar Saparov, we advanced scanning electrochemical microscopy to assess transmembrane water flow (Pohl et al. 1997). The approach is similar to that described in the previous paragraph. Instead of pH sensitivity, we relied on cation-selective microelectrodes. Using these, we detected the dilution of specific ions in the near-membrane water layers induced by transmembrane water flux (Fig. 1A). Such measurements of ion concentration as a function of the distance to the membrane allow for the accurate determination of membrane permeability to water. Notably, this technique works equally well with profiles recorded from within both the water-donating and water-receiving compartment of the planar bilayer.

The approach enabled demonstrating the excellent selectivity of aquaporins. In collaboration with 2003’s Nobel Prize winner Peter Agre, we showed that no more than one in 10^9^ transport events facilitated by orthodox aquaporins is electrogenic (Saparov et al. 2001; Pohl et al. 2001). Later, we also characterized aquaglyceroporins which, despite the larger cross-section of their lumen, also do not conduct ions (Saparov et al. 2005).

In a related study, we focused on water transport through ion channels. We found that the bacterial potassium-selective channel KcsA conducts water well (Saparov and Pohl 2004). The channel’s slow C-type inactivated state precludes only ion conduction, i.e., the inactivated channel represents a selective water channel (Hoomann et al. 2013).

In the latter report (Hoomann et al. 2013), we departed from counting channels by electrophysiology. Instead, we exploited fluorescence correlation spectroscopy. The optical technique allows counting both activated and inactivated channels in reconstituted bilayers. It also afforded me and my younger colleague, Andreas Horner, an exact count of the number of aquaporins reconstituted into large unilamellar vesicles (Horner et al. 2015)﻿ or giant unilamellar vesicles (Boytsov 2020) Fig. 1B. Eventually, this step was crucial for identifying the primary determinant of unitary water channel permeability (Horner et al. 2015): the number of H-bonds that permeating water molecules may form with residues in the channel walls. That is, the water permeability of pores so narrow that water molecules cannot overtake each other is not determined by geometrical factors (channel length and diameter), which govern the flux through micrometer wide pores (Horner and Pohl 2018b). The subject is gaining importance as single-file channels may afford perfect selectivity for water (Horner and Pohl 2018a). Thus, they appeal as tools for water purification and desalinization. The interested reader may find a scientific letter about the energetics of single-file water transport enclosed in the current issue of *Biophysical Reviews* (Pfeffermann et al. 2021b).

## Proton migration along the membrane surface

Narrow water-filled pores may represent railways for transmembrane proton movement as protons may easily hop along water wires. Channels formed by the pentadecapeptide gramicidin provide an example (Saparov et al. 2000). However, aquaporins can exclude protons by providing barriers in the form of positively charged residues and helical dipoles pointing toward the center of the channel (Saparov et al. 2005; Pohl et al. 2001). In the past, we have been interested in monitoring proton movement along interfacial water molecules (Serowy et al. 2003; Zhang et al. 2012) Fig. 1C. The latter endow protons with a high affinity to the membrane surface (Weichselbaum et al. 2017) and further ensure protons’ high interfacial hopping rate, which exceeds the release rate from titratable residues by orders of magnitude (Springer et al. 2011). The combination of high affinity and high mobility may aid the vital efficiency of proton exchange between proton pumps and ATPases.

## Protein translocation across membranes

The transmembrane proton gradient also plays a role in protein translocation through the bacterial plasma membrane facilitated by the heterotrimeric translocon SecYEG. We established that the closed/resting translocon is impermeable to small ions and water by studying the purified channel reconstituted into planar lipid bilayers (Saparov et al. 2007). What is more, our reductionistic experimental system allowed identifying structural elements crucial for sealing the channel (Saparov et al. 2007). We also found that the channel is voltage-sensitive (Knyazev et al. 2020). This property affects the (undesired) leak of ions and protons during protein translocation (Knyazev et al. 2014). It will be key to understanding the protein translocation mechanisms through SecYEG.

## Membrane mechanics

Driven by the realization that the function of membrane proteins frequently depends on constituents of the embedding medium, i.e., lipids, bilayer mechanics became more and more the focus of my research activities. We set out by exploring the coupling between the two membrane leaflets. Contrary to a wide-spread hypothesis, we found a negligible contribution of interdigitation to interleaflet coupling by observing the friction at the membrane midplane of both short- and long-chain fluorescent lipid analogs (Horner et al. 2013). Instead, lipid undulations exert a noticeable effect on interleaflet coupling (Horner et al. 2009). Stiffer regions from both leaflets attract each other simply because their bending rigidities exclude them from highly bent membrane regions (Galimzyanov et al. 2017). The contribution due to undulations scales with the area of the lipid domain and may thus be insufficient to stabilize nanoscopic lipid domains (lipid rafts). In their case, the line tension between thinner disordered domains and thicker ordered domains provides a significant driving force for registration. The elastic energy stored within the border between domains reaches a minimum when domains from opposing leaflets are in register (Fig. 1D). Both undulations and line tension work hand-in-hand (Saitov et al. 2020).

Lipids with light-sensitive conformations allow switching between lipid-ordered and lipid-disordered domains (Saitov et al. 2020). Concerning mechanosensitive channels, photoswitchable lipids pave the way for gating ion channels by light, which we recently demonstrated on the model channel gramicidin A (Pfeffermann et al. 2021a).

## References

[CR1] Antonenko YN, Denisov GA, Pohl P (1993). Weak acid transport across bilayer lipid membrane in the presence of buffers - theoretical and experimental pH profiles in the unstirred layers. Biophys J.

[CR2] Antonenko YN, Pohl P, Denisov GA (1997). Permeation of ammonia across bilayer lipid membranes studied by ammonium ion selective microelectrodes. Biophys J.

[CR3] Boron WF, Endeward V, Gros G, Musa-Aziz R, Pohl P (2011). Intrinsic CO2 permeability of cell membranes and potential biological relevance of CO2 channels. Chemphyschem.

[CR4] Boytsov D, Hannesschlaeger C, Horner A, Siligan C, Pohl P (2020). Micropipette aspiration-based assessment of single channel water permeability. Biotechnol J.

[CR5] Friedman R, Khalid S, Aponte-Santamaria C, Arutyunova E, Becker M, Boyd KJ, Christensen M, Coimbra JTS, Concilio S, Daday C, van Eerden FJ, Fernandes PA, Graeter F, Hakobyan D, Heuer A, Karathanou K, Keller F, Lemieux MJ, Marrink SJ, May ER, Mazumdar A, Naftalin R, Pickholz M, Piotto S, Pohl P, Quinn P, Ramos MJ, Schiott B, Sengupta D, Sessa L, Vanni S, Zeppelin T, Zoni V, Bondar A-N, Domene C (2018). Understanding conformational dynamics of complex lipid mixtures relevant to biology. J Membr Biol.

[CR6] Galimzyanov TR, Kuzmin PI, Pohl P, Akimov SA (2017). Undulations drive domain registration from the two membrane leaflets. Biophys J.

[CR7] Galimzyanov TR, Molotkovsky RJ, Bozdaganyan ME, Cohen FS, Pohl P, Akimov SA (2015). Elastic membrane deformations govern interleaflet coupling of lipid-ordered domains. Phys Rev Lett.

[CR8] Hannesschlaeger C, Horner A, Pohl P (2019). Intrinsic membrane permeability to small molecules. Chem Rev.

[CR9] Hannesschlaeger C, Pohl P (2018). Membrane permeabilities of ascorbic acid and ascorbate. Biomolecules.

[CR10] Hoomann T, Jahnke N, Horner A, Keller S, Pohl P (2013). Filter gate closure inhibits ion but not water transport through potassium channels. Proc Natl Acad Sci USA.

[CR11] Horner A, Akimov SA, Pohl P (2013). Long and short lipid molecules experience the same interleaflet drag in lipid bilayers. Phys Rev Lett.

[CR12] Horner A, Antonenko YN, Pohl P (2009). Coupled diffusion of peripherally bound peptides along the outer and inner membrane leaflets. Biophys J.

[CR13] Horner A, Pohl P (2018a) Comment on “Enhanced water permeability and tunable ion selectivity in subnanometer carbon nanotube porins”. Science 359(6383) 10.1126/science.aap9173 10.1126/science.aap9173 29599215

[CR14] Horner A, Pohl P (2018). Single-file transport of water through membrane channels. Faraday Discuss.

[CR15] Horner A, Zocher F, Preiner J, Ollinger N, Siligan C, Akimov SA, Pohl P (2015). The mobility of single-file water molecules is governed by the number of H-bonds they may form with channel-lining residues. Sci Adv.

[CR16] Knyazev DG, Kuttner R, Bondar AN, Zimmerman M, Siligan C, Pohl P (2020) Voltage sensing in bacterial protein translocation. Biomolecules 10 (1). 10.3390/biom10010078 10.3390/biom10010078 PMC7023257 31947864

[CR17] Knyazev DG, Winter L, Bauer BW, Siligan C, Pohl P (2014). Ion conductivity of the bacterial translocation channel SecYEG engaged in translocation. J Biol Chem.

[CR18] Mathai JC, Missner A, Kugler P, Saparov SM, Zeidel ML, Lee JK, Pohl P (2009). No facilitator required for membrane transport of hydrogen sulfide. Proc Natl Acad Sci USA.

[CR19] Missner A, Kugler P, Antonenko YN, Pohl P (2008) Passive transport across bilayer lipid membranes: Overton continues to rule. Proc Natl Acad Sci USA 105(52):E123. 10.1073/pnas.0809606106 10.1073/pnas.0809606106 PMC2634895 19116282

[CR20] Missner A, Kugler P, Saparov SM, Sommer K, Mathai JC, Zeidel ML, Pohl P (2008). Carbon dioxide transport through membranes. J Biol Chem.

[CR21] Pfeffermann J, Eicher B, Boytsov D, Hannesschlaeger C, Galimzyanov TR, Glasnov TN, Pabst G, Akimov SA, Pohl P (2021a) Photoswitching of model ion channels in lipid bilayers. J Photochem Photobiol B: Biol:112320. 10.1016/j.jphotobiol.2021.112320 10.1016/j.jphotobiol.2021.112320 34600201

[CR22] Pfeffermann J, Goessweiner-Mohr N, Pohl P (2021b) The energetic barrier to single-file water flow through narrow channels. Biophys Rev. 10.1007/s12551-021-00875-w 10.1007/s12551-021-00875-w PMC8724168 35035593

[CR23] Pohl EE, Peterson U, Sun J, Pohl P (2000). Changes of intrinsic membrane potentials induced by flip-flop of long- chain fatty acids. Biochemistry.

[CR24] Pohl P, Antonenko YN, Rosenfeld EH (1993). Effect of ultrasound on the pH profiles in the unstirred layers near planar bilayer lipid membranes measured by microelectrodes. Biochim Biophys Acta.

[CR25] Pohl P, Saparov SM (2000). Solvent drag across gramicidin channels demonstrated by microelectrodes. Biophys J.

[CR26] Pohl P, Saparov SM, Antonenko YN (1997). The effect of a transmembrane osmotic flux on the ion concentration distribution in the immediate membrane vicinity measured by microelectrodes. Biophys J.

[CR27] Pohl P, Saparov SM, Antonenko YN (1998). The size of the unstirred layer as a function of the solute diffusion coefficient. Biophys J.

[CR28] Pohl P, Saparov SM, Borgnia MJ, Agre P (2001). Highly selective water channel activity measured by voltage clamp: analysis of planar lipid bilayers reconstituted with purified AqpZ. Proc Natl Acad Sci USA.

[CR29] Saitov A, Akimov SA, Galimzyanov TR, Glasnov TN, Pohl P (2020). Ordered lipid domains assemble via concerted recruitment of constituents from both membrane leaflets. Phys Rev Lett.

[CR30] Saparov SM, Antonenko YN, Koeppe RE, Pohl P (2000). Desformylgramicidin: a model channel with an extremely high water permeability. Biophys J.

[CR31] Saparov SM, Antonenko YN, Pohl P (2006). A new model of weak acid permeation through membranes revisited: does Overton still rule?. Biophys J.

[CR32] Saparov SM, Erlandson K, Cannon K, Schaletzky J, Schulman S, Rapoport TA, Pohl P (2007). Determining the conductance of the SecY protein translocation channel for small molecules. Mol Cell.

[CR33] Saparov SM, Kozono D, Rothe U, Agre P, Pohl P (2001). Water and ion permeation of aquaporin-1 in planar lipid bilayers. Major differences in structural determinants and stoichiometry. J Biol Chem.

[CR34] Saparov SM, Pohl P (2004). Beyond the diffusion limit: water flow through the empty bacterial potassium channel. Proc Natl Acad Sci USA.

[CR35] Saparov SM, Tsunoda SP, Pohl P (2005). Proton exclusion by an aquaglyceroprotein: a voltage clamp study. Biol Cell.

[CR36] Serowy S, Saparov SM, Antonenko YN, Kozlovsky W, Hagen V, Pohl P (2003). Structural proton diffusion along lipid bilayers. Biophys J.

[CR37] Springer A, Hagen V, Cherepanov DA, Antonenko YN, Pohl P (2011). Protons migrate along interfacial water without significant contributions from jumps between ionizable groups on the membrane surface. Proc Natl Acad Sci USA.

[CR38] Weichselbaum E, Österbauer M, Knyazev DG, Batishchev OV, Akimov SA, Nguyen TH, Zhang C, Knör G, Agmon N, Carloni P, Pohl P (2017). Origin of proton affinity to membrane/water interfaces. Sci Rep.

[CR39] Zhang C, Knyazev DG, Vereshaga YA, Ippoliti E, Nguyen TH, Carloni P, Pohl P (2012). Water at hydrophobic interfaces delays proton surface-to-bulk transfer and provides a pathway for lateral proton diffusion. Proc Natl Acad Sci USA.

[CR40] Zocher F, Zeidel ML, Missner A, Sun TT, Zhou G, Liao Y, von Bodungen M, Hill WG, Meyers S, Pohl P, Mathai JC (2012). Uroplakins do not restrict CO2 transport through urothelium. J Biol Chem.

